# Realised Thermal Niches in Marine Ectotherms Are Shaped by Ontogeny and Trophic Interactions

**DOI:** 10.1111/ele.70017

**Published:** 2024-12-03

**Authors:** Alaia Morell, Yunne‐Jai Shin, Nicolas Barrier, Morgane Travers‐Trolet, Bruno Ernande

**Affiliations:** ^1^ IFREMER, Unité halieutique Manche Mer du Nord Ifremer, HMMN Boulogne sur mer France; ^2^ MARBEC, Univ. Montpellier, Ifremer, CNRS, IRD Sète/Montpellier France; ^3^ Puget Sound Institute University of Washington Tacoma Tacoma WA USA; ^4^ DECOD (Ecosystem Dynamics and Sustainability), L'Institut Agro, IFREMER, INRAE Nantes France

**Keywords:** aerobic scope, bioenergetic trophic web model, fish, food limitation, hypoxia, life stage

## Abstract

Understanding the response of marine organisms to temperature is crucial for predicting climate change impacts. Fundamental physiological thermal performance curves (TPCs), determined under controlled conditions, are commonly used to project future species spatial distributions or physiological performances. Yet, real‐world performances may deviate due to extrinsic factors covarying with temperature (food, oxygen, etc.). Using a bioenergetic marine ecosystem model, we evaluate the differences between fundamental and realised TPCs for fish species with contrasted ecology and thermal preferences. Food limitation is the primary cause of differences, decreasing throughout ontogeny and across trophic levels due to spatio‐temporal variability of low‐trophic level prey availability with temperature. Deoxygenation has moderate impact, despite increasing during ontogeny. This highlights the lower sensitivity of early life stages to hypoxia, which is mechanistically explained by lower mass‐specific ingestion at older stages. Understanding the emergence of realised thermal niches offers crucial insights to better determine population's persistence under climate warming.

## Graphical abstract

Understanding marine organisms' response to temperature is crucial for correctly predicting climate change impacts. Using a multispecies bioenergetic model as a virtual laboratory to compare fundamental and realised thermal performance curves (TPCs), we find that realised TPCs differ the most from fundamental ones for early life stages and lower trophic level species, that is, the smallest organism. This provides valuable insights for predicting species distribution with metabolism traits under climate warming.

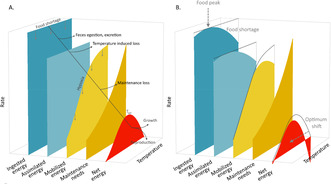

## 1 Introduction

Temperature has a profound impact on biological systems, from cellular (Koch et al. 2021) to ecosystem scales (Parmesan 2006), which is of significant concern in the context of climate change. Temperature directly affects the physiological rates (Gillooly et al. 2002), ecology (Clarke 2019) and biogeography (Deutsch, Penn, and Seibel 2020) of marine ectotherms. Changes in individual physiology and bioenergetics affect different levels of ecological organisation, directly influencing individual growth, reproduction and survival (Audzijonyte et al. 2018) and indirectly impacting demographic rates, species interactions and overall community structure (Beaugrand and Kirby 2018; Payne et al. 2021). Understanding individual physiological responses to temperature is gaining attention, as it is critical to projecting future marine population and community dynamics under climate change (Audzijonyte et al. 2018; Lefevre 2016; Lefevre, McKenzie, and Nilsson 2017; Rose et al. 2024).

**FIGURE 1:**
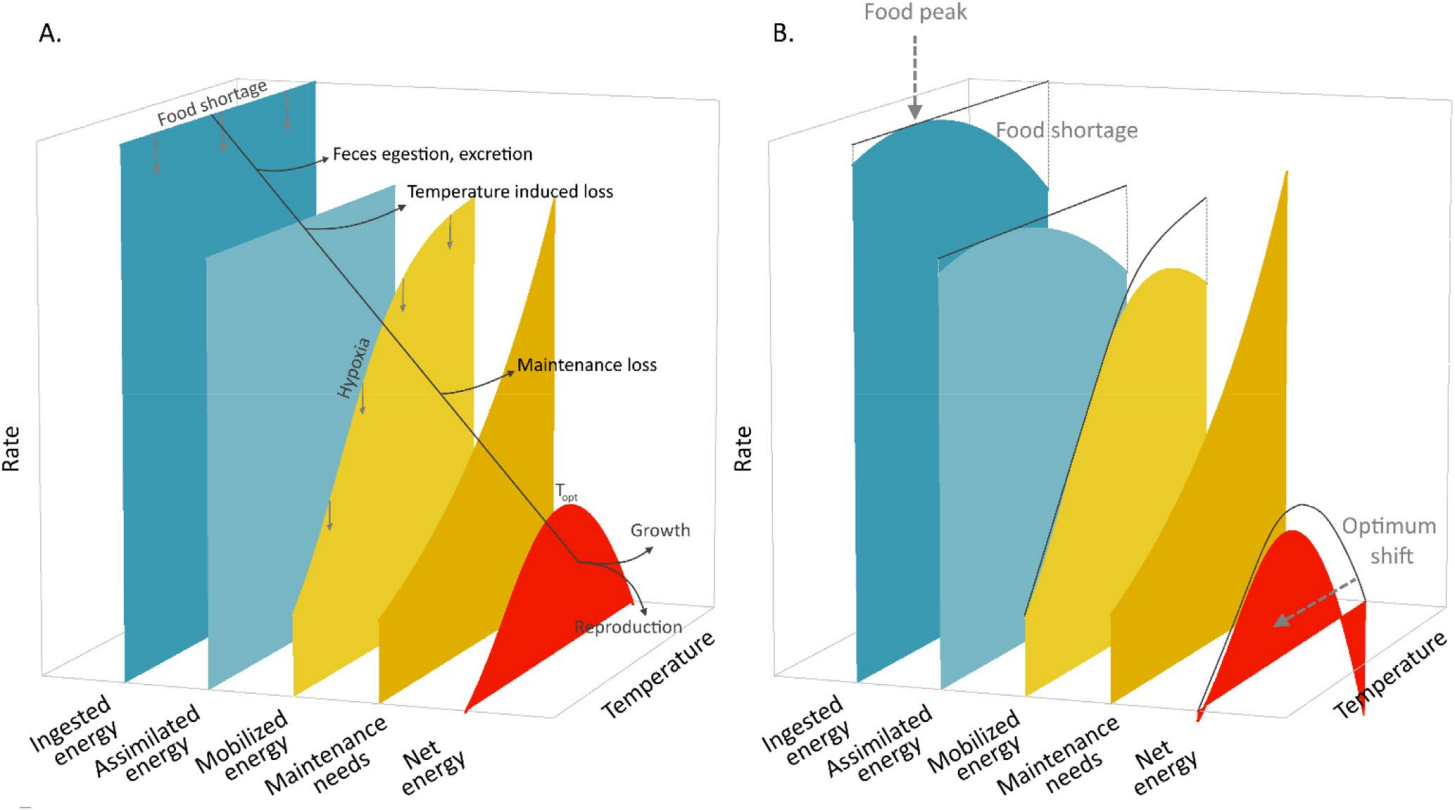
Thermal of bioenergetic fluxes from ingestion to tissue growth, as modelled in our framework. (A) The net energy (~aerobic scope; red curve) for growth and reproduction is the difference between mobilised energy (~maximum metabolic rate; yellow curve) and maintenance costs (~resting metabolic rate; orange curve). Because energy mobilisation increases with temperature more slowly than maintenance needs, the *fundamental* (i.e. under maximum ingestion and normoxia) net energy thermal performance curve conforms to the expected dome shape. Food shortage (downward grey arrows) may impact ingested energy (dark blue curve) and its assimilated fraction (light blue curve) available for mobilisation and thus all downstream fluxes (Figure S1). Hypoxia (downward grey arrows) may impact energy mobilisation (yellow curve) and thus all downstream fluxes (Figure S1). (B) Extrinsic factors, such as food abundance, may vary with temperature and impact ingested energy and the downstream bioenergetic fluxes. The red curve here depicts a *realised* net energy TPC when food abundance, and hence ingestion, varies with temperature. The *realised* net energy TPC optimum corresponds to a lower value than the *fundamental* one. Furthermore, the *realised* TPC amplitude and width are lower.

Aerobic scope, the difference between maximum and resting metabolic rate (Fry 1971), represents an aquatic ectotherm's energy available for all energy expenditure beyond maintenance. Typically, its response to temperature is characterised by a dome‐shaped thermal performance curve (TPC) due to the slower increase in maximum oxygen supply with temperature compared to resting demand (Jutfelt et al. 2024) (Figure 1A). Because the aerobic scope measures physiological performance, its TPC is frequently used as a proxy for ectotherms' thermal niches and their biological rate TPCs, including bioenergetics, growth, reproduction or fitness (Pörtner and Peck 2010).

Utilising aerobic scope as a framework to predict aquatic ectotherms' performance and fitness responses to temperature is debated (Clark, Sandblom, and Jutfelt 2013; Jutfelt et al. 2018; Lefevre, McKenzie, and Nilsson 2017; Lefevre, Wang, and McKenzie 2021). Aerobic scope is just one permissive factor among other constraints, including food intake and oxygen saturation, that determine the net energy available for physiological processes (Jutfelt et al. 2018). Relying solely on aerobic scope to predict thermal niches may be misleading, as these extrinsic factors may also vary with temperature (Figure 1). Extrinsic factors explain the differences between an ectotherm's *fundamental* thermal niche or TPC, defined as the temperature range supporting physiological functioning under resting conditions with positive net energy regardless of other factors (Figure 1A), and its *realised* niche, which accounts for both intrinsic (e.g. physiology) and extrinsic factors (e.g. species interactions) (Pörtner, Bock, and Mark 2017, Figure 1B).

Extrinsic factors that reduce upstream fluxes (e.g. ingestion and energy mobilisation, see Figure 1) uniformly across temperatures can alter the realised TPC of net energy compared to the fundamental TPC, as maintenance needs remain constant (Figure 1A and Figure S1) (Huey and Kingsolver 2019; Vinton and Vasseur 2022). The realised TPC then has lower amplitude, a narrower temperature range and an optimum shifted to lower temperatures. Co‐variation of upstream fluxes with temperature can further reshape the realised TPC (Figure 1B), generating indirect temperature effects via extrinsic factors. These indirect effects remain understudied, especially in ecosystemic settings where multiple extrinsic factors including species, trophic levels or physical conditions co‐vary with temperature over space and time.

Recent theoretical studies highlight that realised TPCs of population growth are affected by temperature‐dependent prey dynamics (Vinton and Vasseur 2022). Within aquatic ecosystems, similar effects could arise from temperature‐dependent trophic interactions due (i) the temperature response of predators and prey distribution and (ii) temperature‐driven fluctuations in their abundances. Reduced oxygen levels are also expected to impact aquatic organisms (Laffoley and Baxter 2019), potentially affecting their realised TPCs due to temperature and oxygen saturation co‐variation.

Using the bioenergetic marine ecosystem model Bioen‐OSMOSE (Morell et al. 2023), this study investigates how trophic interactions and oxygen saturation determine the differences between fundamental and realised TPCs of net energy available for metabolic processes. Bioen‐OSMOSE is an individual‐based model that represents the spatiotemporal dynamics and bioenergetics of multiple high‐trophic level species, primarily fish, in regional marine ecosystems. It is forced by temperature, oxygen and low‐trophic level prey fields. Trophic interactions emerge from opportunistic size‐based predation between co‐occurring individuals. Life‐history processes arise mechanistically from bioenergetics and vary with temperature and oxygen. This study investigates (i) the difference between individual‐level fundamental and population‐level realised net energy TPCs; (ii) how this difference depends on species, life stages and their characteristics (spatial distribution, life history, trophic ecology) and (iii) the main ecological determinants of this difference (prey abundance, competition, predation, oxygen saturation).

## 2 Materials and Methods

### 2.1 Model Description

#### 2.1.1 General Description of Bioen‐OSMOSE

To understand how temperature directly and indirectly affects net energy in high trophic level marine species, we use the application of the Bioen‐OSMOSE model to the North Sea‐Eastern English Channel ecosystem (Morell et al. 2023) as a ‘virtual laboratory’. Bioen‐OSMOSE represents spatially explicit, age‐ and size‐structured high trophic level species dynamics as they emerge from individuals' life cycle. It explicitly describes individuals' bioenergetics from food ingestion to somatic and gonadic tissue production and their responses to oxygen, temperature and food variations. Bioenergetics determine high trophic level species' life‐history traits: somatic growth rate, maturation age and size, absolute fecundity, starvation mortality and, in conjunction with opportunistic size‐based predation, predation mortality. Therefore, population and community dynamics result from the mechanistic individual‐level life cycle modelling, species' spatial distribution and predator–prey interactions.

Individuals' bioenergetics follow a biphasic growth model (Andersen 2019; Boukal et al. 2014; Quince et al. 2008), allocating body mass‐dependent energy fluxes between maintenance, somatic growth and gonadic growth to capture physiological trade‐offs (Roff 1993; Stearns 1992). Sexual maturation is modelled through maturation reaction norms, depicting maturation plastic responses to body growth variations (Heino, Dieckmann, and Godø 2002; Stearns and Koella 1986). This combination mechanistically describes somatic growth, sexual maturation and reproduction as emerging from the energy fluxes sustained by size‐based opportunistic predation. Additionally, energy mobilisation and maintenance costs depend on temperature, resulting in a dome‐shaped TPC of net energy available for tissue production, whose height, width and position are modulated by the impact of oxygen on energy mobilisation.

The model's biological unit is a school (a super‐individual in individual‐based modelling). Each school *i* comprises individuals of the same species, born simultaneously and biologically identical, sharing individual‐level state variables (age, somatic mass, gonadic mass, abundance, spatial location and taxonomic identity) at each time step *t*.

#### 2.1.2 Details of Some Key Bioenergetic Processes in Bioen‐OSMOSE

**FIGURE 2:**
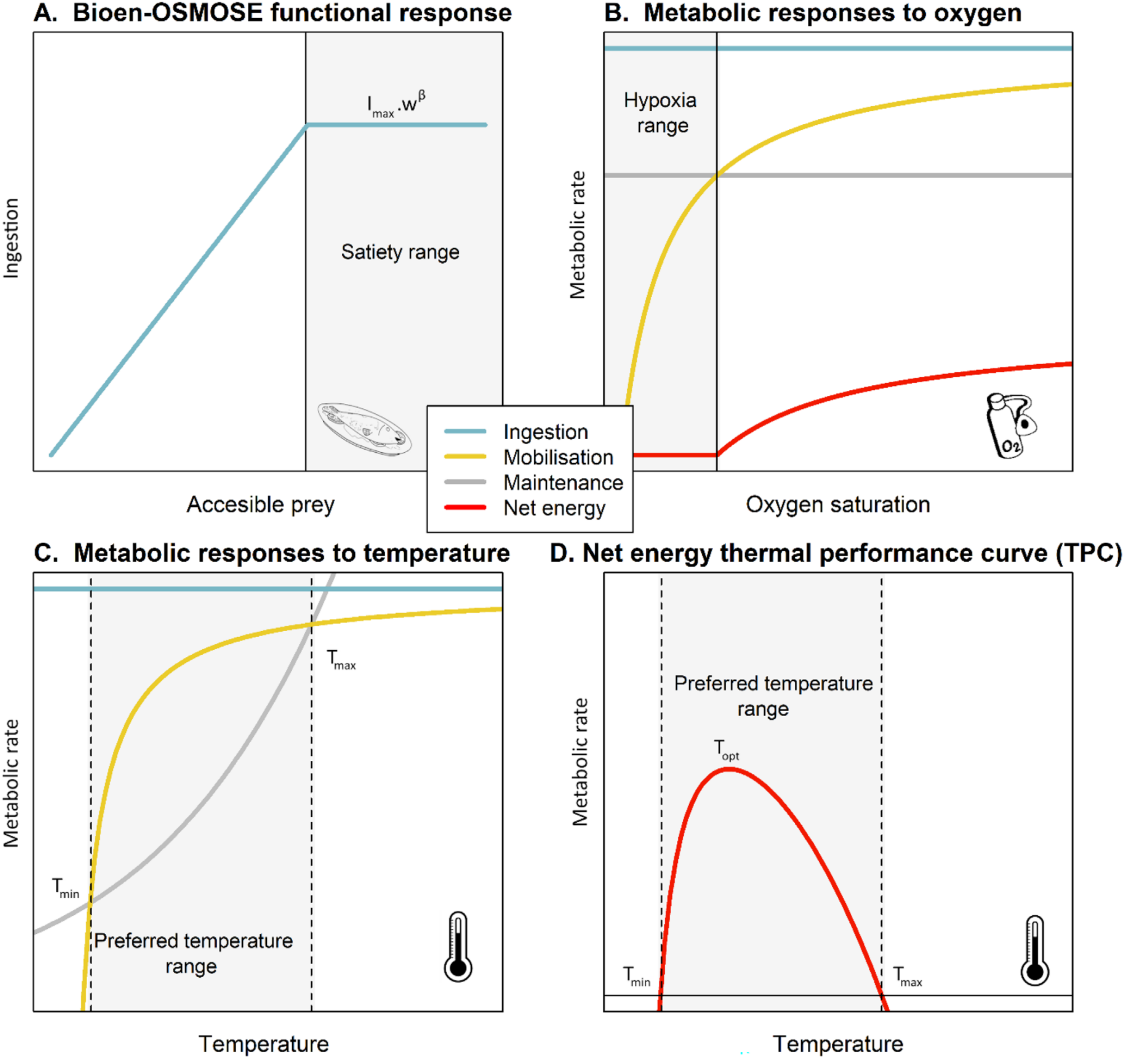
(A) Functional response in Bioen‐OSMOSE, that is, ingested energy as a function of accessible prey biomass. When accessible prey biomass is above the satiety threshold (vertical line), ingestion is constant (grey area) and scales with somatic mass *w* with the mass‐specific maximum ingestion rate *I*_max_ as scaling coefficient. (B) Metabolic response to oxygen. The range of values where maintenance needs are greater than mobilisation supply is the hypoxic range, shown in grey. (C) Metabolic response to temperature. Values in the range where maintenance needs are lower than mobilisation supply is the preferred temperature range, in grey. (D) The net energy available for tissue production responds to temperature according to a dome‐shaped thermal performance curve. This response and the preferred temperature range emerge from differences in mobilisation and maintenance response, described in (C).

Details of the model and its assumptions are available in Morell et al. (2023) and key equations for the present paper in Supporting Information S2. Essential bioenergetic processes for this study are presented hereafter and illustrated in Figures 1 and 2.

The ingested energy rate *I*(*i*, *t*) (Equation S1) follows a Holling's type 1 functional response with a plateau: it increases linearly with accessible prey biomass *P*(*i*, *t*) until reaching a maximum, *I*_max_ *w*(*i*, *t*)^*β*^, reflecting satiety and scaling with individual somatic mass *w*(*i*, *t*), where the maximum mass‐specific ingestion rate *I*_max_ is the scaling coefficient (Figure 2A). Accessible prey biomass comprises prey that (i) falls within the relevant prey‐size range determined by *w*(*i*, *t*) and (ii) co‐occurs spatiotemporally with the school *i*, which depends on the spatial distributions of both predator and prey (i.e. low trophic level prey fields and other high trophic level schools moving according to Brownian motion in their distribution area). During the early‐life stage, *I*_max_ is set 1.4–1.9 times higher, depending on the species, to depict faster mass‐specific growth during this period (Equation S2).

Ingested energy is partly assimilated, with the remainder lost through excretion and egestion. A fraction of the assimilated energy is then mobilised, depending on temperature and oxygen conditions as informed by ecophysiology experiments (Equation S3), to cover tissue maintenance and growth. Energy mobilisation converts nutrients into usable ATP using oxygen (Clarke 2019) Hence, its rate *E*_m_(*i*, *t*) (or ‘maximum metabolic rate’ in ecophysiology; Deutsch et al. 2015), increases with dissolved oxygen saturation following a dose–response function (yellow curve, Figure 2B, Equation S4) until a maximum is set by assimilated energy. It also rises with temperature following Arrhenius law due to chemical reaction rate acceleration until a temperature threshold above which it plateaus or declines due to capacity limitations to sustain oxygen uptake and delivery for ATP production or enzyme denaturation (yellow curve, Figure 2C and Equation S5; Pawar, Dell, and Savage 2015). Mobilised energy fuels metabolic processes, starting in priority with maintenance costs *E*_m_(*i*, *t*) (Equation S6) (or ‘resting metabolic rate’ in ecophysiology; Deutsch et al. 2015), which increases with temperature following Arrhenius law (Gillooly et al. 2002; Kooijman 2010) (grey curve, Figure 2C and Equation S7). The net energy rate for tissue production *E*_P_(*i*, *t*) is then the difference between mobilised energy *E*_M_(*i*, *t*) and maintenance *E*_m_(*i*, *t*) (red curve Figure 2D and Equation S8).

All else being equal, the mobilised energy rate *E*_M_ increases with temperature more slowly than the maintenance rate *E*_m_. As a result, the net energy rate *E*_P_ response to temperature is dome‐shaped, with an optimal temperature *T*_opt_ that maximises net energy and a range of temperatures yielding positive net energy bounded by minimal and maximal preferred temperature, *T*_min_ and *T*_max_, reflecting the *fundamental* TPC (red curve Figure 2D). However, net energy *E*_P_ also depends on ingested energy *I*, which is influenced by available prey biomass *B* and dissolved oxygen saturation [O_2_] through the mobilised energy rate *E*_M_. Hence, when these factors covary with temperature, the *realised* TPC of the net energy rate *E*_P_ deviates from the *fundamental* one. The net energy rate *E*_P_(*i*, *t*) is considered as a performance indicator as it fuels somatic growth before maturation and contributes to both somatic and gonadic growth after maturation, directly affecting organism fitness.

### 2.2 Application of Bioen‐OSMOSE to the North Sea‐Eastern English Channel Ecosystem

This study applies Bioen‐OSMOSE to the North Sea‐Eastern English Channel ecosystem (Bioen‐OSMOSE‐NS) to simulate the dynamics of the upper part of the food web under the forcing of temperature, oxygen and low trophic level dynamics. The study area spans a wide latitudinal range from 49° N to 62° N in northwestern Europe, including the North Sea (excluding the Norwegian trench, i.e. depths beyond 200 m) and the Eastern English Channel. Bioen‐OSMOSE‐NS covers approximately 570,000 km^2^ with a 30 km × 30 km regular grid of 632 cells and a temporal resolution of 15 days.

A full description of Bioen‐OSMOSE‐NS, its parameterisation and its calibration is available in Morell et al. (2023). Explicitly representing 16 high trophic level species, it is parameterised and calibrated to reflect a steady‐state of the ecosystem in terms of fisheries landings (ICES database) (ICES 2019), assessed species' biomass (for assessed species) and high trophic level species' length‐at‐age averaged over the period 2010–2019. Bioen‐OSMOSE‐NS is forced by a 15‐day climatology representing spatial and seasonal variability in low trophic level species biomass, temperature and dissolved oxygen saturation for an average year between 2010 and 2019 based on the regional coupled physical‐biogeochemical POLCOMS‐ERSEM model outputs (Butenschön et al. 2016). At every time step and in each cell, the biomass of two phytoplankton, three zooplankton and three benthos groups from POLCOMS‐ERSEM and two homogeneous benthos groups is supplied as potential low trophic level prey for the high trophic level species. Biomass is vertically integrated for the plankton groups while benthic groups are found only at the bottom. Similarly, vertically integrated temperature and dissolved oxygen saturation are used to force pelagic and demersal high trophic level species bioenergetics while bottom values are used for benthic high trophic level species. The 16 high trophic level species modelled in Bioen‐OSMOSE‐NS are composed of one shrimp functional group and 15 teleost fish species including five small pelagic, seven demersal and three flatfish species.

This study uses Bioen‐OSMOSE‐NS as a virtual laboratory to investigate the determinants of realised net energy TPC curve by focusing on the 15 teleost fish species. The fundamental net energy TPCs were shaped by species' thermal preferences with ingestion, mobilisation, maintenance and their responses to temperature parameterised to reproduce the characteristic points (*T*_min_, *T*_opt_, *T*_max_) of the fundamental TPC for each species (Figure 2D). *T*_min_ and *T*_max_ (Table S3) were informed by the database of Dahlke et al. (2020), which includes values estimated from experimental data for some species (2 of our species) supplemented by values inferred from phylogeny for others (9 species). A linear model was fitted between *T*_min_ and *T*_max_ from climatic niches (Drira et al. 2023) and the physiological *T*_min_ and *T*_max_ from the database for the 11 available species and was used to estimate physiological *T*_min_ and *T*_max_ for 4 species not available in the database. No experimental data were available to estimate fundamental *T*_opt_, so it was derived from species global distribution models, following Drira et al. (2023). These models use occurrence data from across the species' entire geographic range, capturing both thermal tolerance limits (marginal populations) and their optimum (core populations). Therefore, they approximate fundamental thermal niche, unlike regional models that reflect realised niches (Araújo and Peterson 2012). Moreover, geographic range boundaries in aquatic ectotherms closely match experimental thermal tolerance limits (Dahlke et al. 2020; Sunday, Bates, and Dulvy 2012). An estimate of species' average mass‐specific net energy acquisition based on Age‐Length data from scientific surveys (NS‐IBTS‐Q1, North Sea International Bottom Trawl Survey (2010–2019), available online at https://datras.ices.dk) under the specific temperature conditions in the North Sea further informed the fundamental TPC curve. The full method is detailed in Morell et al. (2023).

### 2.3 Simulations and Processing of the Outputs

Results were obtained by running the calibrated configuration for 80 years, with a 70‐year spin‐up period to reach steady‐state, with the last 10 years used for analysis. To construct realised TPCs of net energy rate *E*_P_ for each life stage and species, we averaged *E*_P_ across all schools of the same life stage within each cell at each time step. The average was recorded with the corresponding vertically integrated (pelagic and demersal life stage) or bottom (benthic life stage) temperature in the cell. The resulting dataset of spatiotemporal co‐variation of the average *E*_P_ and temperature was used to construct the realised net energy TPC for each species and life stage. Three life stages were considered: early life (from larvae mouth opening to 1 year), juvenile stage (1 year to sexual maturation) and adult (after sexual maturation). The realised net energy TPC, denoted by $E_{O_{2},B}\left(T\right)$, reflects variation in available food biomass B and dissolved oxygen saturation $\left[O_{2}\right]$ across temperatures, while the fundamental TPC, denoted by $E_{∙,∙}\left(T\right)$ (where a dot as subscript indicates independence), and was constructed as the response of $E_{P}$ to temperature under optimal food and oxygen conditions (maximum ingestion and 100% dissolved oxygen saturation).

The deviation of the realised TPC $E_{O_{2},B}\left(T\right)$ from the fundamental one $E_{∙,∙}\left(T\right)$ was quantified as their mean relative difference across all data points for a given life stage: $D_{O_{2},B}=\frac{1}{n}\sum_{j=1}^{n}\frac{E_{∙,∙}\left(T_{j}\right)-E_{O_{2},B}\left(T_{j}\right)}{E_{∙,∙}\left(T_{j}\right)}$, where the number of data points n is the cumulative number of cells occupied by that life stage over the 10‐year simulation period used for analysis. The sources of this difference, food limitation and oxygen unsaturation, were further disentangled. First, we estimated the realised TPC under optimal food conditions $E_{O_{2},.}\left(T\right)$ using temperature and oxygen values from each cell in the species habitat map for the corresponding life stage. The mean relative difference between $E_{O_{2},.}\left(T\right)$ and the fundamental TPC $E_{∙,∙}\left(T\right)$ quantified the deviation due to oxygen limitation $D_{O_{2},∙}$. Second, energy fluxes being additive in our framework, the remaining difference up to $D_{O_{2},B}$ accounted for the consequence of food limitation, estimating $D_{∙,B}$.

## 3 Results

### 3.1 Comparison of Fundamental and Realised Net Energy TPCs

**FIGURE 3:**
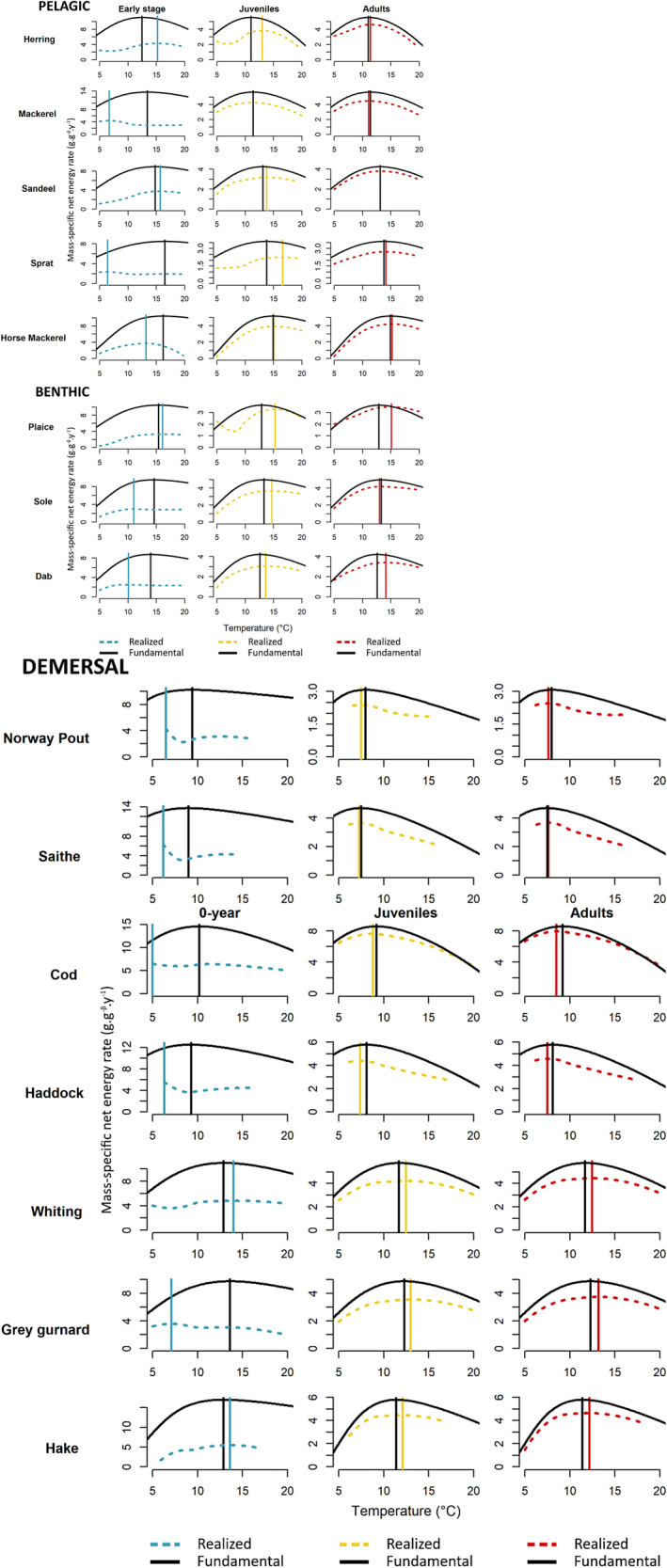
Fundamental and realised net energy TPCs per species for early life stages, juveniles and adults. The maximum ingestion rates (and therefore the TPCs) are different between early life stage and older stages. The fundamental TPC is calculated under optimal food and oxygen conditions and depicted by the black continuous curves. The blue, the yellow and the red dashed curves are the realised TPCs for the early stage, juvenile and adult respectively. Note that realised TPCs depend on the conditions encountered by fish individuals and do not always cover the entire temperature range used for fundamental TPCs. In the North Sea, temperature ranges from 5°C to 20°C. Vertical lines show temperatures at which the net energy rate is highest ($T_{\mathrm{opt}}$).

Realised net energy TPCs differed from fundamental ones for all life stages and species, with the largest deviations observed in early‐life stages (Figure 3). Early‐life stage realised TPCs differed from fundamental ones both in shape (e.g. a ‘V shape’ instead of a dome shape for haddock, Norway pout and saithe) and absolute level for all species. In contrast, adults realised TPCs were similar to fundamental ones in shape and differed mainly in the absolute level of net energy rate. For most species, the shape of juvenile realised TPCs resembled adult ones, except for herring, sprat and plaice.

For all species and all stages, the optimal temperature $T_{\mathrm{opt}}$ maximising the net energy rate along the fundamental TPC, parameterised independently, fell within the temperature range encountered in the NS ecosystem (Table S4). However, there were differences between fundamental and realised $T_{\mathrm{opt}}$, varying among species and life stages (Table S4; Figure 3). Five species had similar fundamental and realised $T_{\mathrm{opt}}$ across life stages: saithe, Norway pout, horse mackerel, haddock and hake (sum of squared differences in Table S4). In contrast, sprat, cod, grey gurnard and dab exhibited the most different realised and fundamental $T_{\mathrm{opt}}$ (Table S4). For 11 out of 15 species, the early‐life stage realised $T_{\mathrm{opt}}$ differed most from the fundamental one (> 2°C difference; Table S4).

### 3.2 Sources of Difference Between Fundamental and Realised Net Energy TPCs

**FIGURE 4:**
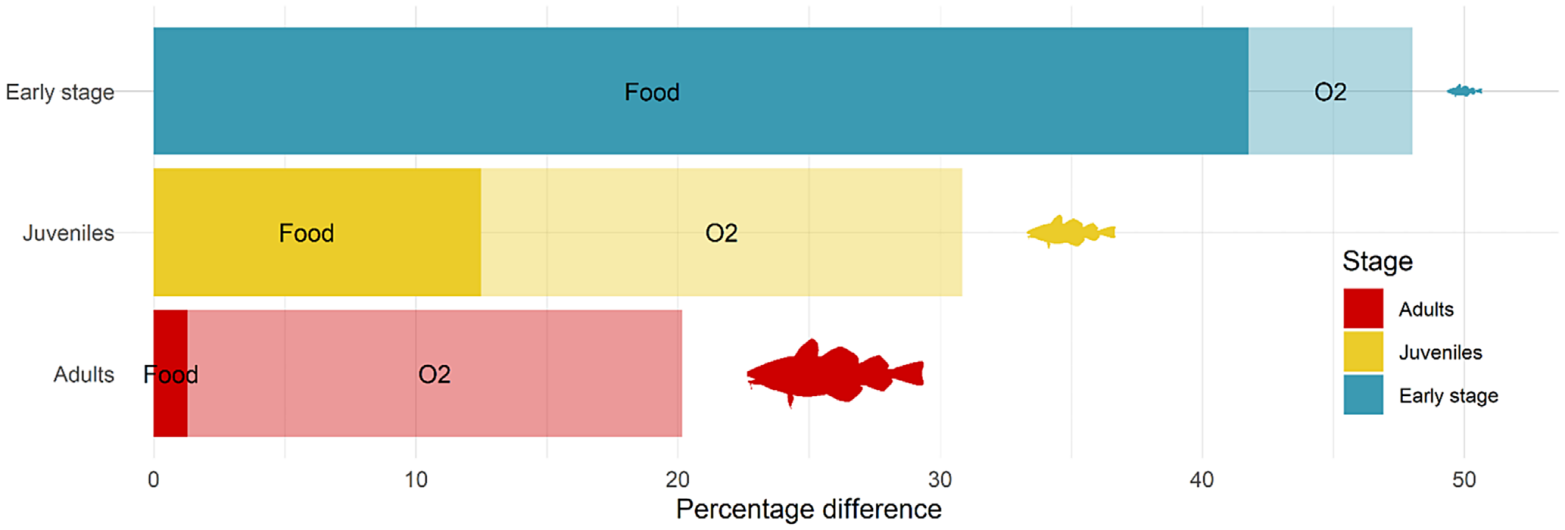
Percentage difference between fundamental and realised net energy TPCs according to life stages, averaged over the 15 species of the model. The stacked bars represent $D_{O_{2},B}$, the percentage difference between the fundamental and the realised $D_{O_{2},B}$ for the three life stages. The light colour part corresponds to the difference due to oxygen limitation $D_{O_{2},∙}$ and the dark colour part is the remaining difference explained by food limitation $D_{∙,B}$.

Oxygen‐unsaturated environment and/or limited food availability impact on the difference between fundamental and realised TPCs and the relative contribution of the two sources depending on life stage exhibited similar patterns across species (Figure 4 and Figure S5). Early‐life stages exhibited the largest differences $D_{O_{2},B}$ between fundamental and realised TPCs, averaging 48% (Figure 4) and ranging between 41.4% and 57.3% depending on species (Figure S5). Juvenile stages displayed intermediate differences, averaging 30.8% with a range of 19.4%–44.8%. Adult stages presented the smallest differences, averaging 20.2% with a range of 13.3%–26.3%. Beyond the lower differences between TPCs for older life stages, the results indicated a shift in the main source of disparity from food limitation to oxygen limitation with age. The difference is due to food limitation, $D_{.;B}$, decreased with age from an average of 41.8% at early‐life stage to 12.5% at juvenile stage to 1.3% at adult stage (Figure 4; corresponding ranges are 34%–51.2%, 1.2%–31.5% and 0%–4.5% respectively; Figure S5). Meanwhile, the difference due to oxygen, $D_{O_{2},∙}$, increased from an average of 6.8% at early‐life stage to 19.3% at juvenile stage to 19.8% at adult stage (Figure 4; ranges 3.6%–8.6%, 8.9%–19.3% and 12.1%–19.8% respectively; Figure S5).

### 3.3 Trophic Level and the Food‐Related Difference Between Fundamental and Realised Net Energy TPCs

**FIGURE 5:**
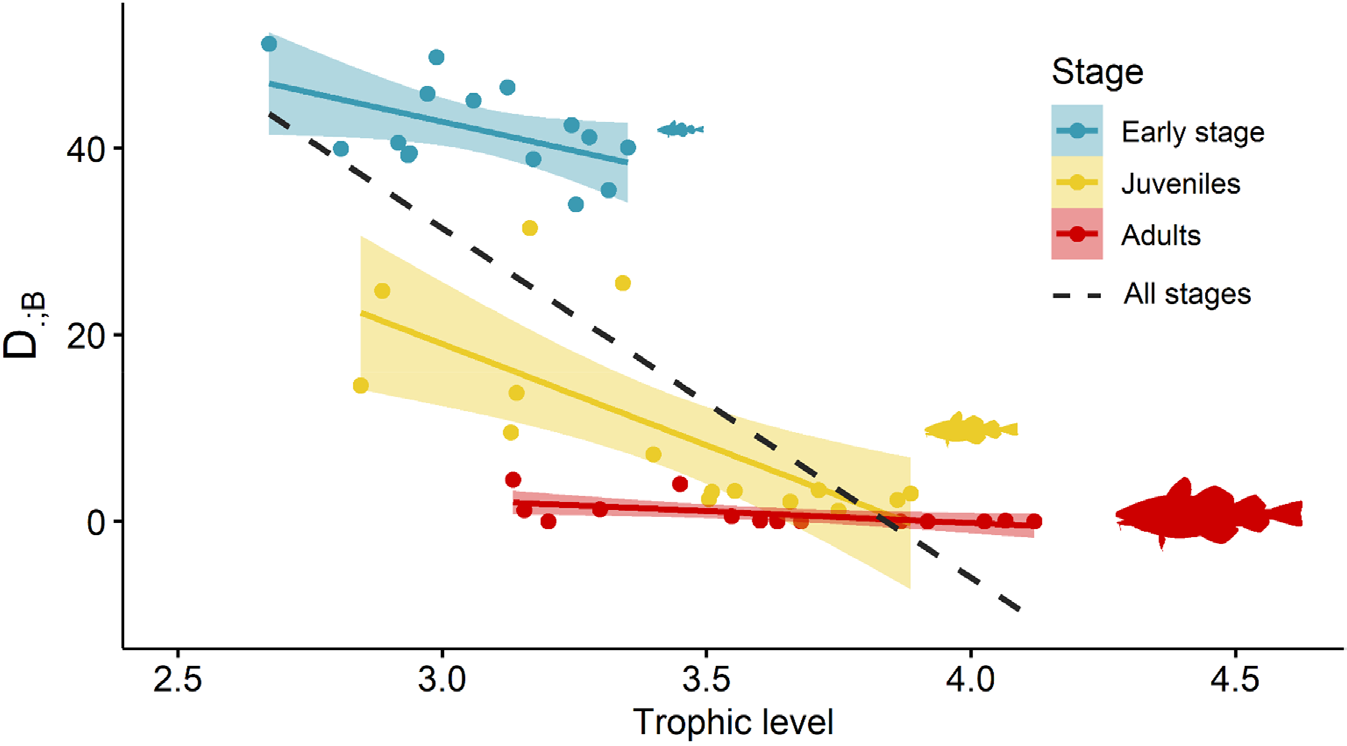
Difference between fundamental and realised net energy rate TPCs due to food limitation, $D_{.;B},$ per life stage as a function of trophic level. The trophic level was estimated per species per life stage from the outputs of Bioen‐OSMOSE. The regression line coefficients are estimated with an ANCOVA model explaining the difference $D_{.;F}$ by trophic level as the continuous covariable and life stage as a categorical factor (see Table S6).

The difference between fundamental and realised net energy TPCs due to food limitation $D_{.;B}$ decreased significantly with species trophic level (Table S6) and tended towards zero for the highest trophic levels (trophic level around 4) (Figure 5). The interaction between trophic level and life stage was significant overall (Table S6), with $D_{.;B}$ response to trophic level varying across life stages, although always in the direction of a decrease. For the three life stages, trophic level had a significant effect on $D_{.;B}$ (Table S6), which decreased by 12.53%, 21.7% and 2.5% per trophic level (TL) for early‐life stage, juveniles and adults respectively. The intercept values decreased with age: the early‐life stage intercept was higher than the juvenile one, which was higher than the adult one (Figure 5), which confirmed that the difference between TPCs due to food limitation decreased with age and ontogeny (see previous subsection and Figure 4).

## 4 Discussion

Fundamental aerobic scope TPCs help understand the temperature response of performance or fitness in aquatic ectotherms (Pörtner, Bock, and Mark 2017) and project population dynamics under climate change scenarios (Deutsch et al. 2008; Pörtner 2021; Pörtner and Farrell 2008; Vasseur et al. 2014). However, understanding how in situ conditions affect realised TPCs is critical to bridge the gap between aerobic scope TPCs estimated under laboratory conditions and their application to wild populations (Jutfelt et al. 2018). Using an individual‐based spatialised trophic web model that incorporates individual‐level bioenergetics, this study introduces a novel approach to investigate how oxygen saturation and food spatiotemporal dynamics generate differences between fundamental and realised TPCs and provides generic insights into the determinants (life stage, trophic level, predator–prey match‐mismatch) of these differences.

The first conclusion of this study is that the realised net energy TPC was always lower than the fundamental one (by 13%–57%, Figure S5), corroborating quantitatively Jutfelt et al.'s (2018) idea that temperature is only a permissive factor of realised performances. Both oxygen unsaturation and food limitation contributed to this difference across all species (Figure 4; Figure S5), which confirms previous studies (Angilletta 2009; Rubalcaba et al. 2020). Notably, the overall difference between realised and fundamental TPCs decreased across ontogeny, while its primary driver shifted from food limitation to oxygen unsaturation. However, the rise in oxygen's contribution only partially compensated for the decline in food limitation, resulting in a net decrease. These ontogenetic changes emphasise the importance of early‐life stages, not least because of their pivotal role in development (Anderson 1988; Cushing 1990; Di Pane et al. 2019; Joly et al. 2021) and recruitment (Olsen et al. 2011; Payne et al. 2009). Earlier studies have shown that early‐life stages have narrower fundamental thermal tolerance (Pörtner and Peck 2010). Our findings further suggest that their realised thermal niche is particularly sensitive to indirect temperature effects mediated by extrinsic factors.

Our findings show that food limitation contribution decreases with increasing trophic level, both across life stages and among species within the same life stage (Figure 5 and Table S5). These patterns are primarily driven by size. In fish, ontogenetic shift in diet and trophic level are strongly driven by size (Sánchez‐Hernández et al. 2019). Similarly, within fish communities, trophic level is positively related to body size (Jennings et al. 2001). Our model predicts diet variation across life stages and size class (Morell et al. 2023), consistent with field data in the area (Pinnegar 2014; Timmerman et al. 2020) and, more generally, with observed ontogenetic diet shifts (Sánchez‐Hernández et al. 2019). By modelling diet as emerging from opportunistic size‐based predation between spatiotemporally co‐occurring individuals, our model effectively captures size‐related ontogenetic shifts in diet and trophic level within species, as well as trophic level variation across species (Morell et al. 2023).

Larger sizes, whether due to ontogeny or species differences, reduce the impact of food limitation on realised TPCs because temperature‐induced spatiotemporal variability in prey abundance decreases as predators' trophic levels increase. Diet variations with ontogeny or across species of different trophic levels may thus explain the contribution of food limitation to the difference between fundamental and realised TPCs. Early‐life stages and low trophic level fish mainly consume phytoplankton and zooplankton, whose abundance exhibits high variability tied to temperature variations (Kunze et al. 2022; Chavez, Messié, and Pennington 2011). There is an indirect bottom‐up effect of temperature on realised TPC through its influence on plankton production. Conversely, adults and high trophic level fish feed on more stable prey against temperature fluctuations, such as macro‐zooplankton for pelagic species and benthic prey or small fish for demersal and benthic species (Morell et al. 2023). As a result, temperature‐related prey abundance variability decreases as predator trophic level increases, whether due to ontogeny or species differences, reducing the probability of prey–predator mismatch and diminishing the role of food limitation in shaping realised TPCs relative to fundamental ones. Since the increase in body size during ontogeny is a characteristic shared by all fish, the ontogenetic effect on realised TPCs can be generalised to other fish communities across aquatic ecosystems (Lawson et al. 2018; Sánchez‐Hernández et al. 2019). Likewise, within size‐structured fish communities where larger body size correlates with higher trophic level, as seen in pelagic communities, realised TPCs are expected to be shaped by trophic level (Jennings et al. 2001).

The deoxygenation effect exhibits the opposite pattern, with its contribution to the difference between realised and fundamental TPCs increasing with ontogeny (Figure 4 and Figure S5). It emerges from the higher maximum mass‐specific ingestion rate of early‐life stages, an assumption originally included to reflect faster growth during the larval and postlarval periods (Figure S7) (Osse and Boogaart 1995). In our model, all else being equal, the net energy rate increases with ingestion rate because mobilised energy scales with ingestion, while maintenance costs do not (Equation S3). With constant maintenance costs (grey line, Figure 2B) and an identical dose–response of energy mobilisation to oxygen (yellow curve, Figure 2B), a given level of oxygen unsaturation causes an identical relative reduction in mobilised energy across ontogenetic stages. However, as ingestion decreases, this same relative reduction results in a larger relative decrease in the net energy rate, which is the difference between mobilised energy and maintenance rate (Figure S7). Since early‐stages have higher maximum mass‐specific ingestion rates than juveniles and adults, their realised TPCs are then less impacted by hypoxia in relative terms. This is consistent with studies showing that the maximum mass‐specific ingestion rate decreases with body mass in fish at the interspecific level (Kiørboe and Hirst 2014) and during development at the intraspecific level (Wuenschel and Werner 2004). It is worth noticing that, in our framework, a higher maximum mass‐specific ingestion rate relies implicitly on a higher maximum mass‐specific rate of oxygen supply (Deutsch et al. 2015) to support increased energy mobilisation. We suggest, therefore, that ontogenetic changes in mass‐specific ingestion rate are accompanied by changes in mass‐specific oxygen supply and likely explain the greater hypoxia tolerance observed in fish early‐life stages in field and experimental studies (Müller, Houben, and Pauly 2023; Verberk et al. 2022).

In adults, oxygen limitation primarily explains the difference between realised and fundamental TPCs (Figure 4), with the difference being constant across temperatures (Figure 3). This emphasises a uniform oxygen effect over both space and time, which is in partly due to the rarity of hypoxic conditions as reported in the North Sea ecosystem (Butenschön et al. 2016; Wakelin et al. 2020). It is also influenced by the shape of the physiological response to oxygen: the dose–response of mobilised energy exhibits a very low slope above the hypoxia range (yellow curve and grey range, Figure 2B). This asymptotic behaviour is meant to reflect the fact that ectotherms generally can sustain oxygen supply under moderate hypoxia by increasing ventilation and delivery, with detrimental effects only arising under severe hypoxia (Jutfelt et al. 2024). As a result, mild hypoxia will not significantly alter the realised TPC shape, but more severe reductions in oxygen can. With marine ecosystems facing increased deoxygenation in the future marine due to (i) warming temperatures, (ii) increased stratification and (iii) eutrophication‐induced hypoxia (Laffoley and Baxter 2019), the consequences on realised TPCs could, respectively, be (i) a shift towards lower net energy rates without modifying their shape, (ii) varying impacts on marine organisms according to their water column position, with benthic species facing greater effects due to stratification and (iii) changes in shape at temperatures observed within hypoxic areas due to eutrophication. The emergence of hypoxic areas will likely be a major factor influencing realised TPCs.

Our findings underscore several implications for assessing the thermal preference and tolerance of marine fish and their use for projecting populations' or communities' responses to future climate scenarios. While laboratory studies provide valuable insights into the physiological bases of thermal preference and tolerance, our results reveal their limitations when applied in situ. The fundamental TPC consistently exceeded the realised one across all species and life stages (Figures 3 and 4). This implies that the fundamental thermal tolerance range $\left[T_{\min},T_{\max}\right]$ overestimates the realised one, with fundamental $T_{\min}$ and $T_{\max}$ being lower and larger, respectively, than their realised counterpart. Moreover, fundamental and realised optimal temperatures $T_{\mathrm{opt}}$ systematically differed due to oxygen conditions and prey availability, although to varying degrees depending on life stage and species. The fundamental $T_{\mathrm{opt}}$ is a closer approximation of the realised one for adults of high‐trophic level species compared to early life stages, because extrinsic factors either are weakly limiting, such as food, or have a constant effect over the temperature range, such as oxygen. However, while adult realised and fundamental TPCs are close under current conditions, the differences may widen as climate change progresses. For example, O'Gorman et al. (2023) suggested that warming could simplify food webs, potentially resulting in a decrease in apex predator trophic levels and thus increasing the disparity between realised and fundamental thermal preference and tolerance.

The discrepancy between fundamental TPCs estimated under controlled conditions and realised ones has consequences for assessing the resilience of populations and communities to future warming. Because the net energy fuels somatic and gonadic growth, its response to temperature determines the response of individual life‐history traits (body growth, age and size at maturation, fecundity), which in turn drive population demographic rates (birth, maturation and death rates) that underlie population and community dynamics (Stearns 1992). Specifically, the overestimation of the thermal tolerance range, with $T_{\max}$ estimated to be larger than its actual realised value, biases the prediction of species local persistence at higher temperature. Likewise, the use of fundamental versus realised temperature optimum, $T_{\mathrm{opt}}$, will produce different predictions of population and community responses to global warming as the net energy rate will be under or overestimated depending on species and life stage. In contrast to predictions that the realised $T_{\mathrm{opt}}$ is always lower than the fundamental one under a uniform incoming energy reduction (Huey and Kingsolver 2019; Vinton and Vasseur 2022) (Figure S1), our study found a higher realised $T_{\mathrm{opt}}$ in nearly half of the cases (i.e. species × life‐stages combinations). This arises because food abundance and/or oxygen saturation vary across temperatures due to spatio‐temporal variability, leading to nonuniform incoming energy restriction across temperatures. Notably, variation of food abundance across temperatures emerges from the respective distribution of predators, prey and competitors, which underscores the importance of a multispecific approach.

Understanding how realised TPCs emerge from fundamental ones is necessary to improve future projections of global warming impacts on marine ectotherm dynamics and biodiversity trajectories. Given the influence of abiotic and biotic factors on individual performances, an integrative framework is needed to understand the direct and indirect effects of temperature on marine ectotherms' bioenergetics and demography. Rubalcaba et al. (2020) have already shown that oxygen and temperature are critical determinants of metabolic performances, ecological niches and climate change impacts in aquatic ectotherms. Our results indicate that trophic interactions and their ontogenetic changes are also key to understanding physiological performance variations with temperature in natural environment and, consequently, climate change impacts. Previous research has underscored the importance of incorporating trophic relationships (Albouy et al. 2014) or physiological information (Talluto et al. 2016) in climate niche models to enhance the projection of species' future spatial distributions under climate change. Here, we advocate for mechanistic modelling frameworks that aim at projecting future population, community and ecosystem dynamics under climate change scenarios to account for both physiology and trophic interactions, recognising their interconnected roles in the response of marine ectotherms to global warming.

## Author Contributions

Yunne‐Jai Shin and Bruno Ernande conceived and supervised the project. Alaia Morell and Yunne‐Jai Shin formalised the study question. Bruno Ernande and Alaia Morell conceived the concepts of the model. Nicolas Barrier and Alaia Morell developed the code and validated the model functioning. Bruno Ernande, Morgane Travers‐Trolet and Alaia Morell parameterised the model. Alaia Morell performed the model simulations. All authors interpreted the model outputs. Alaia Morell wrote the first draft of the manuscript. All authors contributed critically to the revisions of the manuscript and gave final approval for submission.

## Conflicts of Interest

The authors declare no conflicts of interest.

### Peer Review

The peer review history for this article is available at https://www.webofscience.com/api/gateway/wos/peer‐review/10.1111/ele.70017.

## Supporting information


Data S1.

## Data Availability

The Bioen‐OSMOSE model code is available on Github (https://github.com/osmose‐model/osmose/tree/master; code version 4.3.3; commit reference: 8a9aef4). The parameters files of Bioen‐OSMOSE‐NS used to generate the results are deposited on Zenodo (10.5281/zenodo.7636041).
