# Supplementary material for: Realised Thermal Niches in Marine Ectotherms Are Shaped by Ontogeny and Trophic Interactions
Source: Ecol Lett. 2024 Dec 3;27(11):e70017. doi: 10.1111/ele.70017 (PMC11613303; doi:10.1111/ele.70017)
Supplement: Supplementary file 1 — Data S1. [file ELE-27-0-s001.docx]

# Supporting information

**Supporting information S1: Figure S1**: Thermal performance curves of the bioenergetic fluxes from ingestion to tissue growth as modeled in our framework. A uniform reduction of energy input across temperatures (see realized ingested energy, turquoise curve, versus maximum ingestion, dark blue curve) (A), due to reduced food availability or abundance for instance, results in a realized net energy thermal performance curve (red curve) with lower amplitude, a narrower temperature range and an optimum temperature (Topt) shifted towards lower values relative to the fundamental thermal performance curve (thin black curve) (B). An identical effect would have been achieved with a uniform decrease in mobilized energy due to generalized hypoxia for instance.


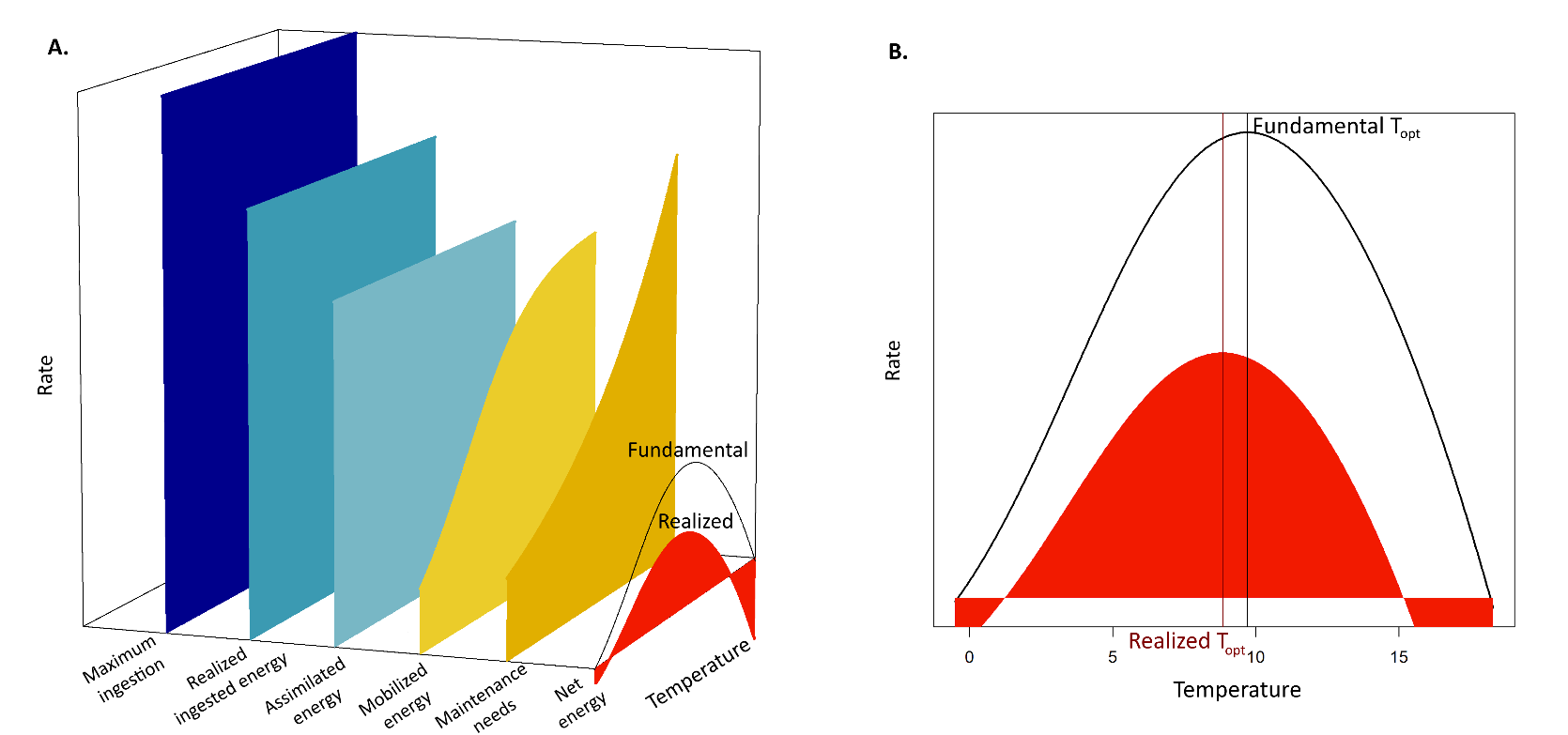


**Supporting information S2: Model equations, adapted from Morell et al. 2023**

### These equations are adapted from Morell *et al.* (2023). The text has been simplified to include only the essential equations necessary to understand the model assumptions relevant to this paper. For a comprehensive justification of the full model, the reader is referred to the original publication.

### Ingestion, assimilation and mobilization

For an individual in school $i$, the ingested food $I(i,t)$ at time step $t$ is described by a Holling’s type 1 functional response:

$I\left( i,t \right)=f\left( B(i,t) \right)=min\left( P\left( i,t \right); I_{max} {\psi\left( i,t \right) w\left( i,t \right)}^{\beta} \right)$ (S1)

with $B\left( i,t \right)$ the prey biomass available to an individual of school $i$, $I_{max}$the maximum ingestion rate per mass unit at exponent$\beta$ (or maximum mass-specific ingestion rate) of individuals in school $i$, $w(i,t)$ their somatic mass, and $\psi\left( i,t \right)$ a multiplicative factor that depends on their life stage such that:

$\psi\left( i,t \right)=\left\{ \begin{matrix} \theta& \text{if} a\left( i,t \right)<a_{l} \\ 1 & \text{otherwise} \end{matrix} \right.$ (S2)

where $a_{l}$ is the age at the end of an early-life fast-growth period (e.g., larval period or larval and post-larval periods, defined according to data availability) and $\theta>1$ is a multiplicative factor accounting for higher mass-specific ingestion rate at this stage. A portion $\xi$ of the ingested food $I\left( i,t \right)$ is assimilated, $(1-\xi)$ being lost due to excretion and feces egestion.

Reserves are not modeled in Bioen-OSMOSE: the assimilated energy is directly mobilized. The difference between assimilated and mobilized energy depends on oxygen and temperature conditions (Fig. 2). Mobilized energy $E_{M}$, referred to as active or maximum metabolic rate in the ecophysiology literature, fuels all metabolic processes such as maintenance, digestion, foraging, somatic growth, gonadic growth, etc… The mobilized energy rate $E_{M}$ is described by:

$E_{M}\left( i,t \right)=\xi I\left( i,t \right) \lambda\left( \left[ O_{2} \right](i,t) \right) \varphi_{M}\left( T(i,t) \right)$ (S3)

where $\lambda\left( \left[ O_{2} \right](i,t) \right)$and $\varphi_{M}\left( T(i,t) \right)$ represent the mobilization responses to dissolved oxygen saturation $\left[ O_{2} \right]\left( i,t \right)=\left[ O_{2} \right](c\left( i,t \right))$ and temperature $T\left( i,t \right)=T\left( c\left( i,t \right) \right)$, respectively, encountered by school $i$. These responses are scaled between 0 and 1, such that under optimal oxygen saturation and temperature conditions, all assimilated energy $E_{M}\left( i,t \right)=\xi I\left( i,t \right)$ is mobilized. In suboptimal conditions, only a fraction of the assimilated energy can be mobilized $E_{M}\left( i,t \right)<\xi I\left( i,t \right)$.

More precisely, the effect of dissolved oxygen arises from the fact that energy mobilization relies on converting the chemical bond energy of nutrients into usable ATP using oxygen (Clarke 2019). This process is described by a dose-response function $\lambda\left( \cdot\right)$ (Thomas *et al.* 2019) which increases with dissolved oxygen saturation:

$\lambda\left( \left[ O_{2} \right] \right)=c_{O,1} \frac{\left[ O_{2} \right]}{\left[ O_{2} \right]+c_{O,2}}$ (S4)

where parameters $c_{O,1} \mathrm{and} c_{O,2}$ represent the asymptote and the slope of the dose-response function. The asymptotic behavior reflects the fact that ectotherms generally can sustain oxygen supply under moderate hypoxia by increasing ventilation and delivery, with detrimental effects only arising under severe hypoxia (Jutfelt *et al.* 2024); Fig. 2B yellow curve). The effect of temperature $\varphi_{M}\left( \cdot\right)$ initially causes energy mobilization to increase with temperature according to an Arrhenius-like law, driven by the acceleration of chemical reaction rates. However, it plateaus or declines at higher temperatures due to limitations in an individual’s ventilation and oxygen delivery capacity (Pörtner 2001), negative temperature-dependence of enzyme-catalyzed chemical reactions (Arcus *et al.* 2016), or even enzyme denaturation (Pawar et al. 2015). The (Johnson & Lewin 1946) model (Pawar et al. 2015) is used to describe this response:

$\varphi_{M}\left( T \right)=\Phi\frac{e^{-\frac{\varepsilon_{M}}{k_{B} T}}}{1+\frac{\varepsilon_{M}}{\varepsilon_{D}-\varepsilon_{M}}e^{\frac{\varepsilon_{D}}{k_{B}}(\frac{1}{T_{p}}-\frac{1}{T})}}$ (S5)

where $k_{B}$ is the Boltzmann constant, $\varepsilon_{M}$ is the activation energy for the Arrhenius-like increase in mobilized energy with temperature $T$ before its peaks at $T_{p}$, and $\varepsilon_{D}$ is the activation energy for the decline in energy mobilization as $T$ increases beyond $T_{p}$. The constant $\Phi=\left( 1+\frac{\varepsilon_{M}}{\varepsilon_{D}-\varepsilon_{M}} \right)e^{\frac{\varepsilon_{M}}{k_{B} T_{p}}}$ is a standardizing factor ensuring that $\varphi_{M}\left( T_{p} \right)=1$.

### Maintenance

The mobilized energy $E_{M}$ fuels all metabolic processes, starting with the maintenance costs of existing tissues $E_{m}$, often referred to as the standard or resting metabolic rate in the ecophysiology literature. The maintenance energy rate $E_{m}$ scales with the individual ’s somatic mass $w(i,t)$ with the same exponent $\beta$ as the maximum ingestion rate and increases with temperature following Arrhenius law (Brown *et al.* 2004; Gillooly *et al.* 2002; Kooijman 2010). It is described as:

$E_{m}(i,t)=c_{m} {w\left( i,t \right)}^{\beta} \varphi_{m}(T(i,t))$ (S6)

where $c_{m}$ is the mass-specific maintenance rate and $\varphi_{m}(\cdot)$ is the Arrhenius function defined as:

$\varphi_{m}\left( T \right)=e^{-\frac{\varepsilon_{m}}{k_{B} T}}$ (S7)

with $\varepsilon_{m}$ the activation energy for the increase of the maintenance rate with temperature.

### Net energy available for new tissue production

The net energy available for new tissues production $E_{P}$ is the difference between the mobilized energy $E_{M}$ and the maintenance costs $E_{m}$ defined as:

$E_{P}\left( i,t \right)=E_{M}\left( i,t \right)- E_{m}\left( i,t \right)$*.* (S8)

All else being equal, since the mobilized energy rate $E_{M}$ increases with temperature more slowly than the maintenance rate $E_{m}$ near the species’ preferred temperature, the emerging relationship between the net energy rate $E_{P}$ and temperature is dome shaped.

**Supporting information S3: Table S3:** Input parameters of the temperature and oxygen responses extracted from ecophysiology experiments for the 15 species detailed in this study and thermal tolerance limits.

|  | **MAINTENANCE** | | | **MOBILIZATION** | | | **THERMAL TOLERANCE LIMITS** | | |
| --- | --- | --- | --- | --- | --- | --- | --- | --- | --- |
|  | **TEMPERATURE** | **SOURCE** | | **OXYGEN** | | **SOURCE** | $\boldsymbol{T}_{\text{min}}$ | $\boldsymbol{T}_{\text{max}}$ | **SOURCE** |
|  | $\varepsilon_{m}$ | $c_{m}$ |  | $c_{O,1}$ | $c_{O,2}$ |  |  |  |  |
| Species | $eV$ | $g\cdot{\Delta t}^{-1}$ |  | *-* | *%* |  | **°C** | **°C** |  |
| Herring (*Clupea harengus)* | 0.60 | 1.49E+11 | Same as sprat | 1.00 | 8.1 | Same as mackerel | -1.3 | 20.1 | Experimental data, Dahlke et al. (2020) |
| Mackerel (*Scomber scombrus)* | 0.33 | 5.03E+06 | Dickson, 2002 | 1.00 | 8.1 | Dickson, 2002 | -0.5 | 24.3 | Phylogenetic inference, Dahlke et al. (2020) |
| Sandeel (*Ammodytes spp)* | 0.47 | 5.40E+08 | Behrens & Steffensen, 2007 | 1.00 | 7.3 | Behrens & Steffensen, 2007 | -0.7 | 27.1 | Phylogenetic inference, Dahlke et al. (2020) |
| Sprat (*Sprattus sprattus)* | 0.27 | 2.57E+05 | Meskendahl, 2013 | 1.00 | 8.1 | Same as mackerel | -0.1 | 24.8 | Phylogenetic inference, Dahlke et al. (2020) |
| Norway pout (*Trisopterus esmarkii)* | 0.17 | 5.82E+03 | Teleost value, Clarke & Johnston, 1999 | 1.48 | 54.6 | Same as cod | 1.4 | 25.1 | Estimated with tolerances from thermal niches |
| Plaice (*Pleuronectes platessa)* | 0.38 | 1.54E+07 | Clarke & Johnston, 1999 | 2.00 | 99.0 | Steffensen *et al.* 1982 | 1 | 26.5 | Phylogenetic inference, Dahlke et al. (2020) |
| Sole (*Solea solea)* | 0.42 | 5.28E+07 | Lefrançois & Claireaux, 2003 | 2.60 | 179.4 | Lefrançois & Claireaux, 2003 | 1.6 | 31.3 | Phylogenetic inference, Dahlke et al. (2020) |
| Saithe (*Pollachius virens)* | 0.26 | 2.74E+05 | Steinhausen et al., 2005 | 1.48 | 54.6 | Same as cod | -0.2 | 21.8 | Phylogenetic inference, Dahlke et al. (2020) |
| Cod (*Gadus morhua)* | 0.48 | 2.07E+09 | Claireaux et al., 2000 | 1.48 | 54.6 | Claireaux et al., 2000 | -2 | 23 | Experimental data, Dahlke et al. (2020) |
| Haddock (M*elanogrammus aeglefinus)* | 0.31 | 1.96E+06 | Average of cod, saithe and whiting rate | 1.48 | 54.6 | Same as cod | -0.9 | 23.3 | Phylogenetic inference, Dahlke et al. (2020) |
| Horse Mackerel (*Trachurus trachurus)* | 0.25 | 1.04E+05 | Geist et al., 2013 | 1.00 | 8.1 | Same as mackerel | 4.8 | 33.5 | Phylogenetic inference, Dahlke et al. (2020) |
| Whiting (*Merlangius merlangus)* | 0.30 | 1.36E+06 | Steinhausen et al., 2005 | 1.48 | 54.6 | Same as cod | 1.1 | 27 | Estimated with tolerances from thermal niches |
| Dab (*Limanda limanda)* | 0.46 | 2.86E+08 | Same as sole | 2.30 | 139.2 | Average of sole and plaice response | 1.8 | 27 | Estimated with tolerances from thermal niches |
| Grey gurnard (*Eutrigla gurnardus)* | 0.31 | 1.47E+06 | Teleost value, Clarke & Johnston, 1999 | 1.48 | 54.6 | Same as cod | 1.5 | 27.7 | Estimated with tolerances from thermal niches |
| Hake (*Merluccius merluccius)* | 0.25 | 1.74E+05 | Average of cod, saithe and whiting rate | 1.48 | 54.6 | Same as cod | 3.7 | 28.9 | Phylogenetic inference, Dahlke et al. (2020) |

**Supporting information S4: Table S4:** Fundamental optimal temperature per species (°C) and its realized counterpart per species and life stage. The last column is the standardized sum of squared differences between the fundamental and the realized for all three stages. The early stage is the life stage for which the realized is the most distant from the fundamental one.

| Species | Fundamental T_opt_ | T_opt_ early stage | T_opt_ juveniles | T_opt_ adult | Sum of squared differences |
| --- | --- | --- | --- | --- | --- |
| Herring | 11 | 15.2 | 13 | 11.4 | 21.8 |
| Mackerel | 11.3 | 6.7 | 11.4 | 11.1 | 21.21 |
| Sandeel | 13.1 | 15.7 | 13.8 | 13.1 | 7.25 |
| Sprat | 13.7 | 6.4 | 16.6 | 14.1 | 61.86 |
| Norway Pout | 8 | 6.5 | 7.5 | 7.6 | 2.66 |
| Plaice | 13.1 | 16.1 | 15.3 | 15.1 | 17.84 |
| Sole | 13.4 | 11 | 14.7 | 13 | 7.61 |
| Saithe | 7.5 | 6.2 | 7.3 | 7.6 | 1.74 |
| Cod | 9.4 | 4 | 8.8 | 8.5 | 30.33 |
| Haddock | 8 | 6.3 | 7.4 | 7.5 | 3.5 |
| Horse Mackerel | 14.9 | 13.2 | 15.1 | 15.2 | 3.02 |
| Whiting | 11.7 | 14 | 12.5 | 12.5 | 6.57 |
| Dab | 12.6 | 10.1 | 13.6 | 14.1 | 27.65 |
| Grey gurnard | 12.2 | 7.1 | 13 | 13.2 | 27.74 |
| Hake | 11.5 | 13.6 | 12.1 | 12.2 | 5.26 |

**Supporting information S5: Figure S5:** Percent difference between fundamental and realized TPC according to life stage and source of difference. For each species, the 3 stacked bars represent the relative difference between the fundamental and the realized TPC $D_{O_{2},B}$ for the three life stages. Limited food and limited oxygen conditions can both contribute to this difference. Therefore, each stacked bar is subdivided into two parts: the light color part corresponds to the difference due to oxygen limitation $D_{O_{2},\cdot}$ and the dark color part is the difference explained by food limitation $D_{\cdot,B}$.


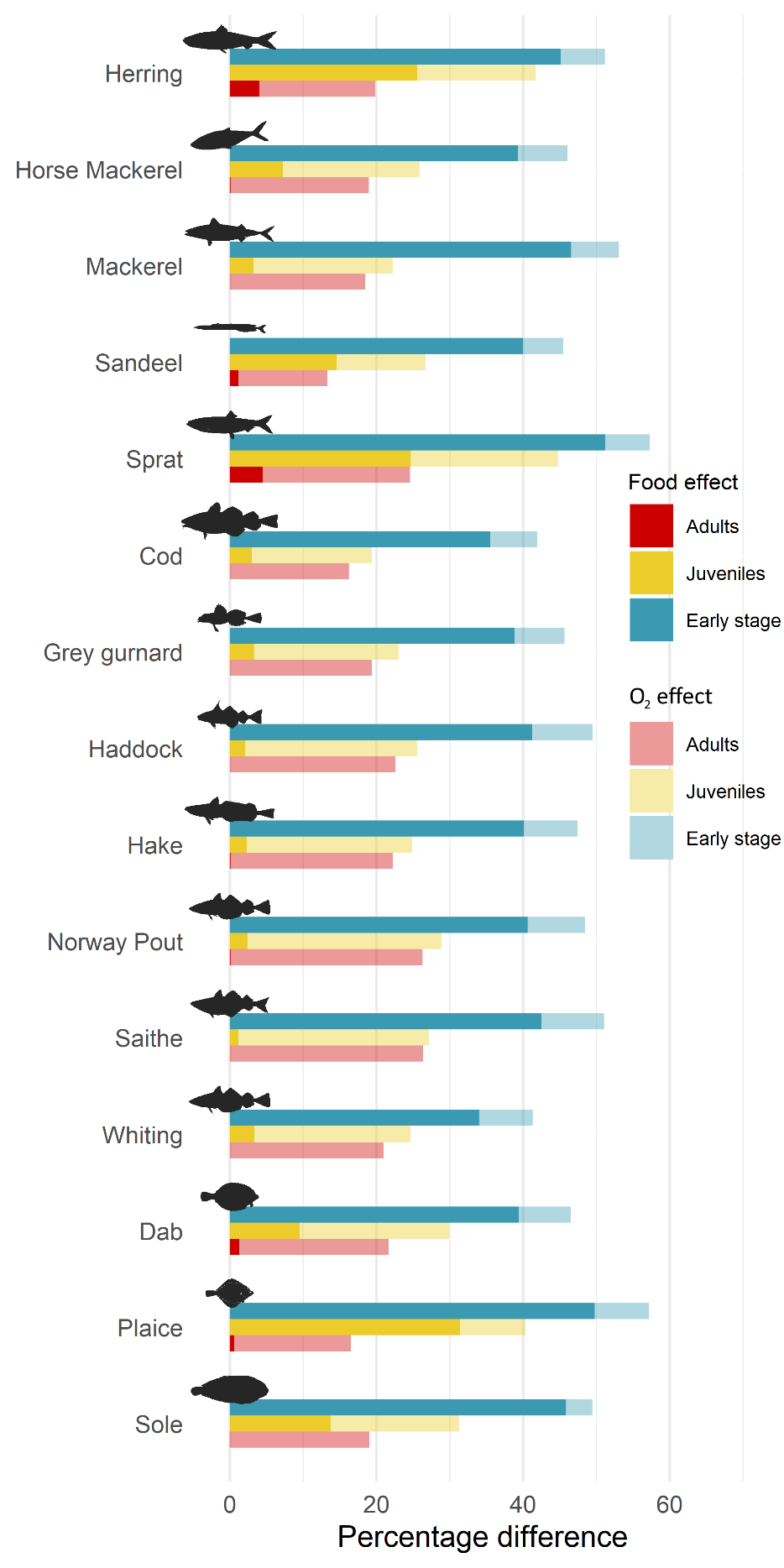


**Supporting information S6: Table S6:** Result of an ANCOVA model of the difference due to food limitation explained by trophic level, life stage, and the interaction between the two variables. (R² = 0.93). Type II F tests for the significance of explanatory variables are presented together with estimated coefficients, their standard error (SE) and significance level (F tests). The residual df is 39. Trophic level was centered around a value of 2 and the coefficients are presented per life stage to help interpretation.

| Explanatory variable | df | F value | Variable P (>F) | Coefficient | Estimate | SE | t values | Coefficient P (>\|t\|) |
| --- | --- | --- | --- | --- | --- | --- | --- | --- |
| Trophic level | 1 | 22.5 | <0.001 | Early-stage slope | -12.5 | 5.8 | -2.2 | 0.05 |
|  |  |  |  | Juvenile slope | -21.7 | 3.9 | -5.5 | <0.001 |
|  |  |  |  | Adult slope | -2.5 | 1 | -2.5 | 0.03 |
| Stage | 2 | 129.1 | <0.001 | Early-stage intercept | 55.4 | 17.8 | 4.5 | <0.001 |
|  |  |  |  | Juvenile intercept | 40.8 | 13.6 | 6.2 | <0.001 |
|  |  |  |  | Adult intercept | 4.9 | 19.8 | -3.7 | 0.01 |
| Trophic level:Stage | 2 | 5.9 | 0.006 |  |  |  |  |  |

**Supporting information S7: Figure S7:** Impacts of a 20% decrease in oxygen on mobilized energy, maintenance net energy, and the difference between the fundamental and realized thermal performance curves (TPCs) for early-stage and adult for a generic fish species. Both groups are parametrized with the same dose-response function for mobilized energy in response to oxygen and temperature, as well as identical maintenance costs in response to temperature. The only difference between the groups is that the mass-specific ingestion rate for the early stage is 1.5 times higher than that of the adult.


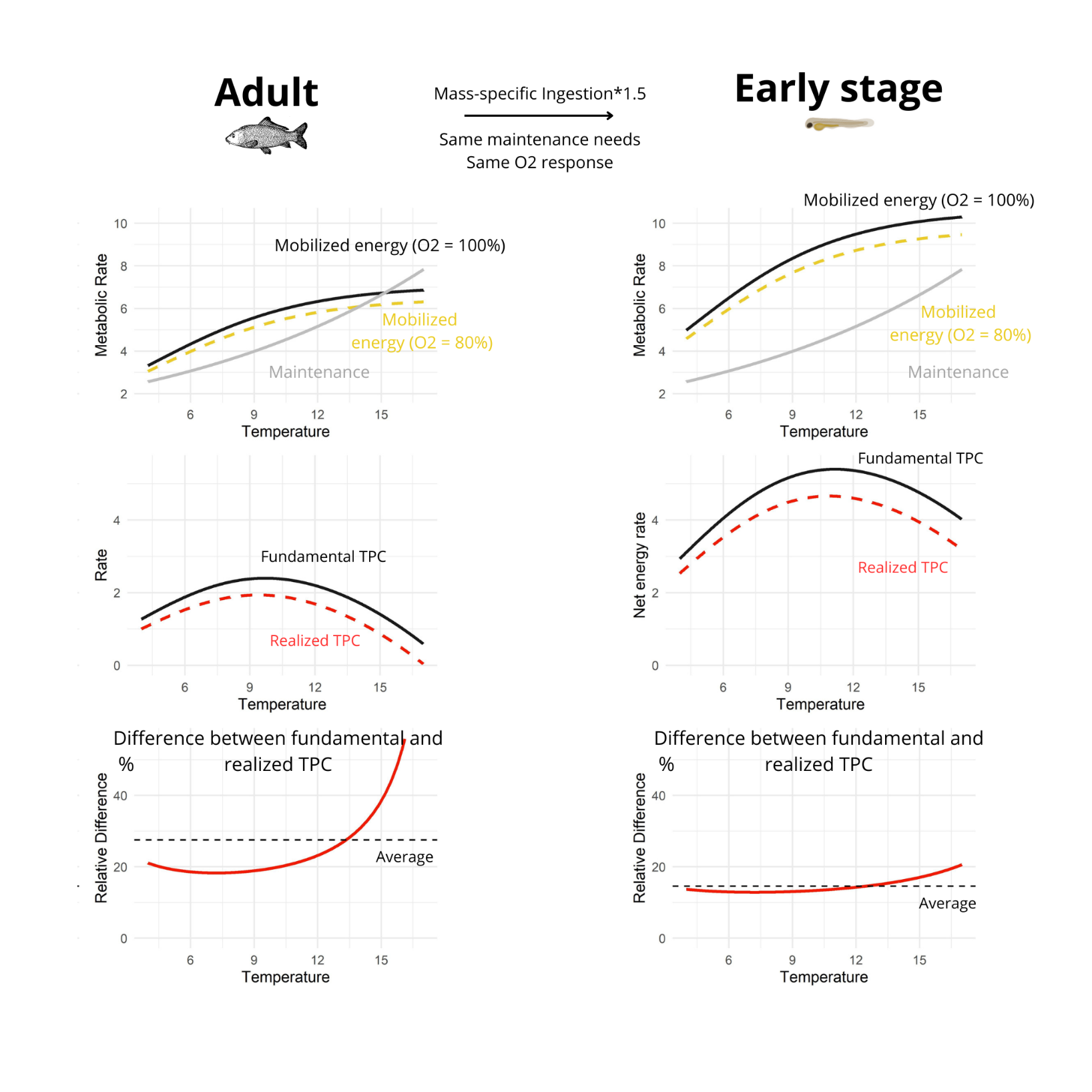


# References

Arcus, V.L., Prentice, E.J., Hobbs, J.K., Mulholland, A.J., Van der Kamp, M.W., Pudney, C.R., *et al.* (2016). On the Temperature Dependence of Enzyme-Catalyzed Rates. *Biochemistry, 55(12), 1681–1688.*

Behrens, J.W. & Steffensen, J.F. (2007). The effect of hypoxia on behavioural and physiological aspects of lesser sandeel, Ammodytes tobianus (Linnaeus, 1785). *Mar Biol*, 150, 1365–1377.

Brown, J.H., Gillooly, J.F., Allen, A.P., Savage, V.M. & West, G.B. (2004). TOWARD A METABOLIC THEORY OF ECOLOGY. *Ecology*, 85, 1771–1789.

Claireaux, G., Webber, D.M., Lagardère, J.-P. & Kerr, S.R. (2000). Influence of water temperature and oxygenation on the aerobic metabolic scope of Atlantic cod (Gadus morhua). *Journal of Sea Research*, 44, 257–265.

Clarke, A. (2019). Energy Flow in Growth and Production. *Trends in Ecology & Evolution*, 34, 502–509.

Clarke, A. & Johnston, N.M. (1999). Scaling of metabolic rate with body mass and temperature in teleost fish. *Journal of Animal Ecology*, 13.

Dahlke, F., Wohlrab, S., Butzin, M. & Pörtner, H.-O. (2020). Experimental data compilation, thermal tolerance and thermal responsiveness of fish species and life stages.

Dickson, K.A. (2002). Temperature effects on sustained swimming in mackerel, 12.

Geist, S.J., Ekau, W. & Kunzmann, A. (2013). Energy demand of larval and juvenile Cape horse mackerels, Trachurus capensis, and indications of hypoxia tolerance as benefit in a changing environment. *Mar Biol*, 160, 3221–3232.

Gillooly, James.F., Charnov, E.L., West, G.B., Savage, V.M. & Brown, J.H. (2002). Effects of size and temperature on developmental time. *Nature*, 417, 70–73.

Johnson, F.H. & Lewin, I. (1946). The growth rate of E. coli in relation to temperature, quinine and coenzyme. *Journal of Cellular and Comparative Physiology*, 28, 47–75.

Jutfelt, F., Ern, R., Leeuwis, R.H.J. & Clark, T.D. (2024). Effects of climate warming. In: *Reference Module in Life Sciences*. Elsevier, p. B978032390801600183X.

Kooijman, S.A.L.M. (2010). Dynamic Energy Budget Theory for Metabolic Organisation. *Cambridge University Press.*

Lefrançois, C. & Claireaux, G. (2003). Influence of ambient oxygenation and temperature on metabolic scope and scope for heart rate in the common sole Solea solea. *Marine Ecology Progress Series*, 259, 273–284.

Meskendahl, L. (2013). Metabolic rates and feeding behaviour of sprat, Sprattus sprattus L. PhD. University of Hamburg, Hamburg, Germany.

Morell, A., Shin, Y.-J., Barrier, N., Travers-Trolet, M., Halouani, G. & Ernande, B. (2023). Bioen-OSMOSE: A bioenergetic marine ecosystem model with physiological response to temperature and oxygen. *Progress in Oceanography*, 103064.

Pawar, S., Dell, A.I. & Savage, V.M. (2015). From Metabolic Constraints on Individuals to the Dynamics of Ecosystems. In: *Aquatic Functional Biodiversity*. Elsevier, pp. 3–36.

Pörtner, H. (2001). Climate change and temperature-dependent biogeography: oxygen limitation of thermal tolerance in animals. *Naturwissenschaften*, 88, 137–146.

Steffensen, J.F., Lomholt, J.P. & Johansen, K. (1982). Gill ventilation and O2 extraction during graded hypoxia in two ecologically distinct species of flatfish, the flounder (Platichthys flesus) and the plaice (Pleuronectes platessa). *Environ Biol Fish*, 7, 157–163.

Steinhausen, M.F., Steffensen, J.F. & Andersen, N.G. (2005). Tail beat frequency as a predictor of swimming speed and oxygen consumption of saithe (Pollachius virens) and whiting (Merlangius merlangus) during forced swimming. *Marine Biology*, 148, 197–204.

Thomas, Y., Flye-Sainte-Marie, J., Chabot, D., Aguirre-Velarde, A., Marques, G.M. & Pecquerie, L. (2019). Effects of hypoxia on metabolic functions in marine organisms: Observed patterns and modelling assumptions within the context of Dynamic Energy Budget (DEB) theory. *Journal of Sea Research*, 143, 231–242.
